# Sex dependent effect of maternal e-nicotine on F1 *Drosophila* development and airways

**DOI:** 10.1038/s41598-021-81607-8

**Published:** 2021-02-24

**Authors:** Natalia El-Merhie, Arne Krüger, Karin Uliczka, Stephanie Papenmeier, Thomas Roeder, Klaus F. Rabe, Christina Wagner, Hanna Angstmann, Susanne Krauss-Etschmann

**Affiliations:** 1 Division of Experimental Asthma Research, Early Life Origins of Chronic Lung Disease, Research Center Borstel, Leibniz Lung Center, German Center for Lung Research (DZL) and the Airway Research Center North (ARCN), Borstel, Germany; 2 Invertebrate Models, Priority Area Asthma & Allergy, Research Center Borstel, Leibniz Lung Center, Borstel, Germany; 3 Department of Molecular Physiology and Zoology, Christian Albrechts University, German Center for Lung Research (DZL) and the Airway Research Center North (ARCN), Kiel, Germany; 4 Department of Pneumology, LungenClinic, German Center for Lung Research (DZL) and the Airway Research Center North (ARCN), Grosshansdorf, Germany; 5 Department of Medicine, Christian Albrechts University, German Center for Lung Research (DZL) and the Airway Research Center North (ARCN), Kiel, Germany; 6 Institute for Experimental Medicine, Christian Albrechts University, German Center for Lung Research (DZL) and the Airway Research Center North (ARCN), Kiel, Germany

**Keywords:** Diseases, Disease model

## Abstract

E-cigarettes are heavily advertised as healthier alternative to common tobacco cigarettes, leading more and more women to switch from regular cigarettes to ENDS (electronic nicotine delivery system) during pregnancy. While the noxious consequences of tobacco smoking during pregnancy on the offspring health are well-described, information on the long-term consequences due to maternal use of e-cigarettes do not exist so far. Therefore, we aimed to investigate how maternal e-nicotine influences offspring development from earliest life until adulthood. To this end, virgin female *Drosophila melanogaster* flies were exposed to nicotine vapor (8 µg nicotine) once per hour for a total of eight times. Following the last exposure, e-nicotine or sham exposed females were mated with non-exposed males. The F1-generation was then analyzed for viability, growth and airway structure. We demonstrate that maternal exposure to e-nicotine not only leads to reduced maternal fertility, but also negatively affects size and weight, as well as tracheal development of the F1-generation, lasting from embryonic stage until adulthood. These results not only underline the need for studies investigating the effects of maternal vaping on offspring health, but also propose our established model for analyzing molecular mechanisms and signaling pathways mediating these intergenerational changes.

## Introduction

Electronic cigarettes (e-cigarettes) have become increasingly popular and their use has increased worldwide. E-cigarettes are heavily advertised with the claim to support smoking cessation and as an healthier and safer alternative to regular tobacco cigarettes^1–3^. This marketing strategy obviously targets highly vulnerable populations such as adolescents and pregnant women^1,4^. The deleterious consequences of tobacco smoking during pregnancy on the future respiratory health of offspring are well-described^5,6^. In contrast, information on the long-term consequences on the biology and health of offspring effects and due to maternal use of e-cigarettes is still scarce^7–9^.

The only study currently available that collected questionnaire data on smoking and e-cigarettes in pregnant women^10^ suggests that the use of e-cigarettes during pregnancy in 248 women causes fetal growth restriction, which in turn is a proposed risk factor for impaired lung function development and respiratory hyper-reactivity^11–13^.

Beyond this report no other epidemiological investigations linking e-cigarette use during pregnancy to offspring health exist so far. In addition, the frequent dual use of tobacco and e-cigarettes complicates the interpretation of human studies^4,14^. Thus, misclassification due to reporting bias and the inability of objective markers such as cotinine or CYP1a1-expression to separate the sources of nicotine cannot be excluded with certainty^10^.

Delayed embryo implantation, reduced birth weight, increased neurodevelopmental vulnerability and vascular dysfunction in young offspring has been demonstrated in several mouse models of maternal e-cigarette use^15–19^. Further, it was shown that maternal e-cigarette exposure altered pulmonary global DNA methylation and cytokine expression in the offspring^20^ potentially increasing the risk for lung disease.

Although e-cigarettes lack tar and carbon monoxide gas^21^, fatal cases due to acute lung injury have been reported to the Center of Disease Control and Prevention (CDC). While tetrahydrocannabinol and Vitamin E acetate were suspected as causal agents, the liquids used in e-cigarettes contain nicotine^22^, heavy metals and other substances already known to be associated with adverse health effects^23–26^. Among these; acrolein, formaldehyde, and acetaldehyde, are examples with reported respiratory toxicity^22,23,27–31^. In addition, liquid nicotine can reach concentrations exceeding those of tobacco cigarettes^32–35^.

As the knowledge on the long-term effects of maternal vaping on offspring health is currently limited, we aimed to investigate in a first step how maternal e-nicotine influences offspring growth and development from earliest life until adulthood. In women and all mammalian model systems embryo-fetal development takes place inside the uterus. We therefore decided to use the fruit fly *Drosophila melanogaster* as they deliver embryos whose further development takes place outside of the females’ body. Therefore, this model facilitates tracking of maternal e-nicotine influence on the offspring from their earliest stages of development until adulthood in a sex-specific way, which simplifies intergenerational studies.

## Materials and methods

### Chemicals

Nicotine used for exposure, ( −)-Nicotine ≥ 99% (GC), was purchased from Sigma-Aldrich Chemie GmbH (Germany).

### Drosophila strain and culture

Experiments were carried out with a wild type *Drosophila melanogaster* strain (Canton S). Virgin female flies were isolated under CO_2_ anesthesia 6 h post-eclosion*.* Flies were cultured on a standard cornmeal/molasses/yeast/agar medium and kept in 175 ml vials at a temperature of 25 °C and a relative humidity of 65% to 75% at a 12 h:12 h light:dark cycle. The flies were exposed to e-nicotine at the age of 5-days. All experiments were performed at room temperature (21–23 °C).

### Nicotine exposure

A self-constructed e-nicotine-exposure system consisted of a 175 ml vial with a nylon mesh cloth inserted in the middle in order to divide the vial in half and to protect the animals from heat stress. The upper part was closed with a foam plug while the bottom part was connected to a coiled nichrome wire attached to a power supply (3.7 V, 1.9 A).

Virgin female flies were divided into e-nicotine-exposed and dH_2_O-control groups (100 flies/group). The flies were free of CO_2-_anaesthesia for 5-days prior to further manipulations. Briefly, 10 μl of nicotine (5 mM) or dH_2_O was pipetted on a nichrome coil which was heated for 15 s. Thereafter, the flies were allowed to rest in the exposure chamber for 2 min before being transferred back to their culture vials. The experiment was repeated once per hour for a total of 8 times. Next, e-nicotine-exposed and dH_2_O-control flies (100 flies per group) were transferred onto two individual grapefruit agar (GA) plates where they were mated with male flies of the same age.

### Geotaxis assay

In order to determine the appropriate concentration of e-nicotine for further experiments, a negative geotaxis assay, for the detection of the flies’ locomotory behavior against gravity, was performed. Briefly, 100 age-matched female flies per treatment were divided into 10 flies/group and exposed to either dH_2_O (control) or 1 mM, 5 mM or 10 mM of nicotine. Thereafter, the flies from each group were transferred into 4 vials and gently tapped to the bottom of the column. The distance of 7 cm was marked on the vials and the number of flies (percentage) that were able to climb above the 7 cm mark by 10 s was recorded. The assay was repeated 3 times per group with 1 min rest between the repetitions.

### Determination of oviposition

Eggs collected on (GA) plates within the first 10 h after mating were discarded and the new (GA) plates were placed into an egg collection cage. Eggs laid by treated and control groups were collected within the subsequent 2 h in order to produce synchronized embryos. Then, GA plates were washed with PBS and the eggs were poured into a 70 µl cell strainer for counting. Fecundity was characterized by the percentage of the total number of eggs observed 2 h after mating. Thereafter, eggs were transferred to standard medium and incubated as described above.

### Egg morphology

The mean length of randomly selected eggs (100 eggs/experiment) deposited within 2 h after mating was estimated from the most proximal to the most distal point. Morphological measurements were taken using an OLYMPUS SZX16 stereomicroscope with Olympus cell-Sens Standard imaging software (Olympus cellSens Standard, Version 1.16, Olympus Corporation, Tokyo, Japan).

### Survival rate

In order to determine the percentage of larvae hatching from the eggs and the percentage of pupae-to-eclosed adults, embryos were transferred into vials containing standard cornmeal diet food and allowed to develop into 1st-instar (L1) larvae. Each vial was plugged with cotton and maintained under conditions of controlled temperature (25 °C) and humidity (60%). After 24 h, cohorts of hatched F1 L1 larvae were counted under the OLYMPUS SZX16 stereomicroscope. In order to study the transgenerational effect of nicotine on the percentage of adult fly eclosion (the ratio of pupae-to-adult), we counted the number of emerged adults i.e. according to the number of pupae the percentage of eclosed adults was calculated in each group.

### Determination of the size and weight of L1 and L3 generation

L1 and L3 larvae (20 larvae/experiment) were extracted from the vials 24 and 72 h, respectively, following egg deposition. Thereafter, the larvae were fixed in 70% ethanol, dried and then either weighed or placed on glass slides for further microscopic analysis. The measurement of the length of L1 and L3 larvae obtained from egg batches laid by e-nicotine- and water-exposed females was carried out from the most proximal to the most distal point of the body with an OLYMPUS SZX16 stereomicroscope using Olympus cell-Sens Standard imaging software (Olympus cellSens Standard, Version 1.16, Olympus Corporation, Tokyo, Japan). The weights of L3 larvae were recorded using Sartorius BP61-scale.

### Tracheal length measurement

L3 larvae (10 to 15 flies) were removed from the medium with tweezers, washed in 1 × PBS and placed on a drop of glycerin on a slide. The slide was then placed on a heating plate at 70 °C for 10 s in order to kill the larvae. Thereafter, the larvae were aligned with the dorsal side facing upwards, covered with a cover-slip and analyzed with an Olympus SZX16 stereomicroscope using cell-Sens Standard imaging software (Olympus cellSens Standard, Version 1.16, Olympus Corporation, Tokyo, Japan). The third thoracic segment of the larvae was measured at a magnification of 20-fold. All images were scaled and saved as Tiff files. These microscopic images were then used to measure the length of secondary branches using ImageJ software (Version 2.1.4.7, Wayne Rasband National Institutes of Health, USA) with NeuronJ plugin (version 1.4.3). First, the images were converted to an 8-bit version and the contrast was increased until the secondary branches were clearly visible (approx. 1.5–2%). All the processed images were saved as tiff files and thereafter analyzed with NeuronJ. Secondary branches were measured using the "tracking" function of NeuronJ. The previously used scaling was used as the basis for the measurement.

### Determination of the length and weight of F1 adult flies

After the eclosion, the first-generation adult female and male flies were maintained as virgins for 5 days before their weight and length was measured. Weights were recorded using a precision scale (Sartorius BP61-scale), whereas the lengths were recorded using and camera-equipped OLYMPUS SZX16 stereomicroscope and analyzed using cell-Sens Standard software (Olympus cellSens Standard, Version 1.16, Olympus Corporation, Tokyo, Japan).

### Survival assay of F1 adult flies

Virgin flies collected within 24-h time-frame were separated by sex immediately after eclosion and transferred into separate 175 ml vials capped by a foam plug. A petri dish containing standard medium was attached to the bottom of the vial. The petri dish was renewed every other day except weekends and the number of dead flies was documented until no flies remained alive. Flies were transferred into new vials every two weeks without CO_2_-anaesthesia.

### Statistical analysis

Values shown are mean ± SEM. The number of samples and the statistical test used for each data set are reported in the corresponding figure legends. Statistical analysis was evaluated by one-way ANOVA, unpaired Student’s t-test and Kaplan–Meier curve, log-rank (Mantel-Cox) in GraphPad Prism version 6.01. The probability of occurrence was selected at p < 0.05. Data show the results obtained from three independent experiments.

## Results

### Dose dependent reduction of *Drosophila* locomotor activity by e-nicotine

To quantify the sensitivity of adult female flies to e-nicotine and to determine the appropriate concentration of e-nicotine to be further used in our experiments, the locomotor behavior of female flies following their exposure to different concentrations of e-nicotine (1, 5 or 10 mM) was measured using a negative geotaxis assay^36^.

**Figure 1:**
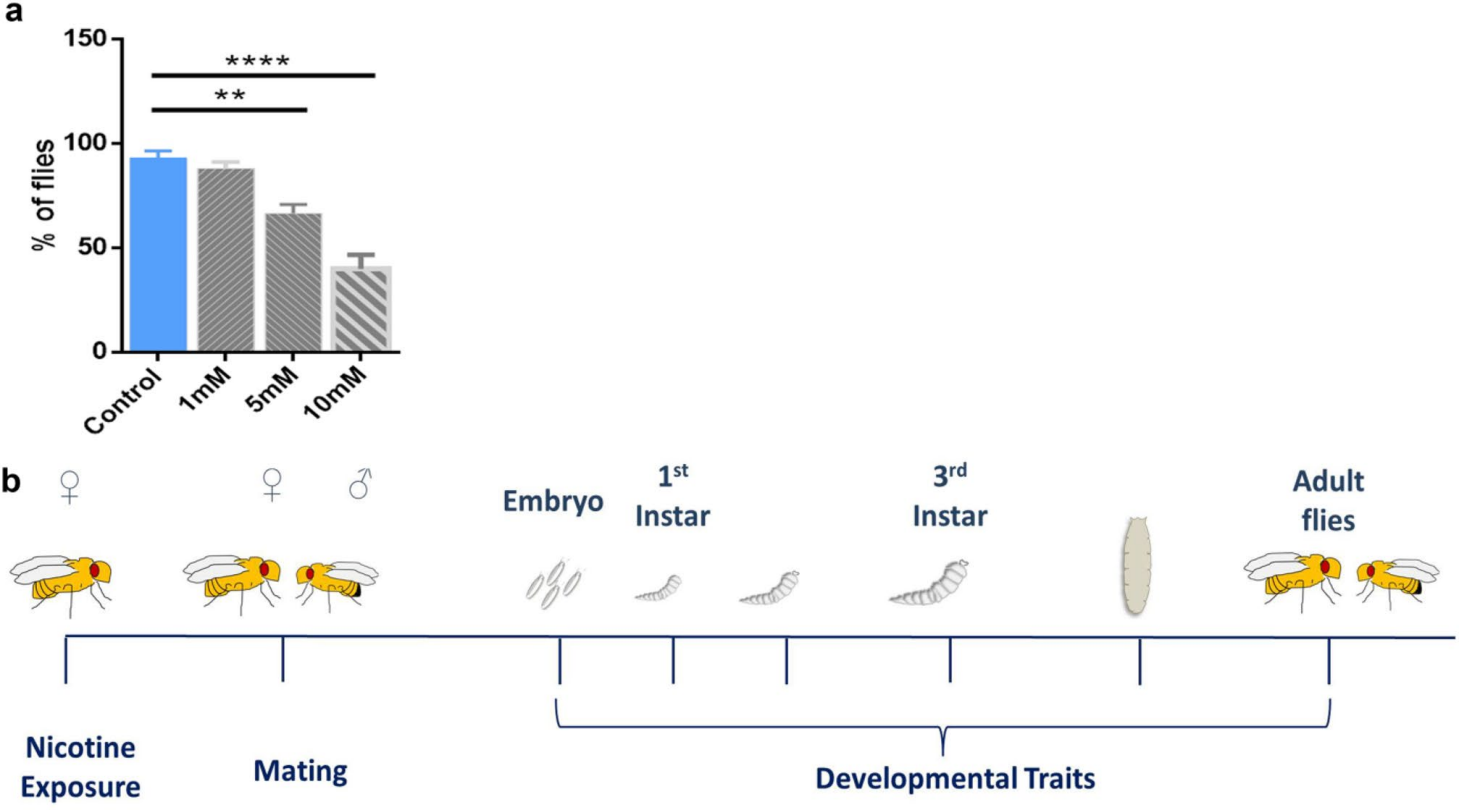
Dose dependent decrease of locomotor activity following e-nicotine exposure. (**a**) The locomotor activity of 10 flies/group vs time. The number (percentage) of flies that reached a height of 7 cm in 10 s following the exposure to different concentrations of nicotine as compared to the water-exposed controls was recorded (**b**) Experimental outline, female flies were exposed to 5 mM of volatilized nicotine for 8 times once per an hour, thereafter, the flies were mated with the males of the same age and their offspring were analysed across the timeline. Values are mean ± SEM, one-way ANOVA, **p ≤ 0.01, ****p ≤ 0.0001, n = 3 independent experiments.

We detected a dose-dependent reduction in the locomotor behavior of female *Drosophila* flies following their exposure to different concentrations of volatilized nicotine. Water-treated and 1 mM e-nicotine-exposed groups exhibited no detectable difference in the climbing behavior where they rapidly climbed back to the top of the column. However, the groups exposed to either 5- or 10-mM of e-nicotine demonstrated a dose-dependent decrease in their climbing behavior, where only 60% and 40% of the flies in each group, respectively, could cross a distance of 7 cm in 10 s (Fig. 1a). In addition, nicotine-volatilization induced hyperactivity and excess grooming behavior at a lower dose and akinesia at the two higher doses. Therefore, a concentration of 5 mM of nicotine was selected for our further experiments since it’s the least concentration of nicotine that interferes with fly’s normal propensity to negatively geotax without leading to a complete loss of flies’ locomotor activity.

Next, to investigate whether maternal exposure to e-nicotine affects the development and the growth of the offspring, female flies were exposed to 5 mM of volatilized nicotine, mated and their offspring were analyzed (Fig. 1b).

### Maternal e-nicotine exposure affects the number and length of the deposited eggs

**Figure 2:**
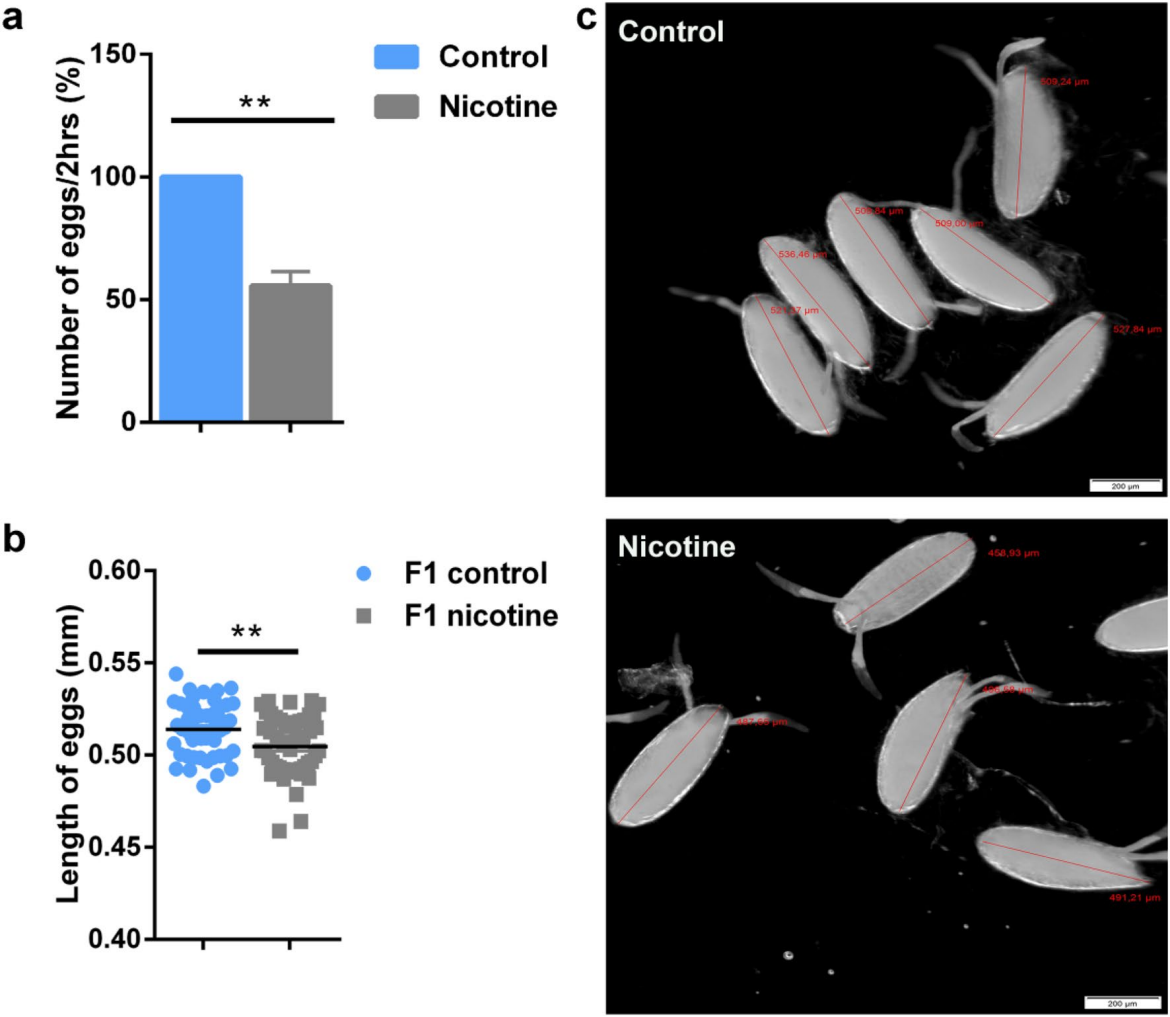
E-nicotine exposure decreases the number and size of the eggs. (**a**) The number of eggs (%) collected per 2 h per group (**b**) Quantification of the egg length (mm) (**c**) Representative stereomicroscope image of the eggs laid on grapefruit agar (GA) per 2 h per group analysed using the image software cellSens (red line represents the measurement of the egg length). Values are mean ± SEM, unpaired t-test, **p ≤ 0.01. Bar represents 200 μm, n = 3 independent experiments.

To analyze whether e-nicotine exposure affects the progeny production of F0 *Drosophila* females, we assessed the reproductive behavior of these flies by counting the number of eggs laid per 2 h. E-nicotine treatment led to a drastic decline in the number of deposited eggs, demonstrating therefore a significant decrease in the fecundity rate by almost 50% in comparison to water-treated controls (Fig. 2a).

Next, to find whether maternal exposure to volatized nicotine affects the size of the laid eggs, the deposited eggs were collected within 2 h and their size was measured. The results show that maternal e-nicotine treatment significantly reduced the length of the eggs by approximately 0.2 mm (P < 0.001) (Fig. 2a right column,c) in comparison to the control group (Fig. 2b left column,c).

### The rate of eggs yielding hatched larvae

**Figure 3:**
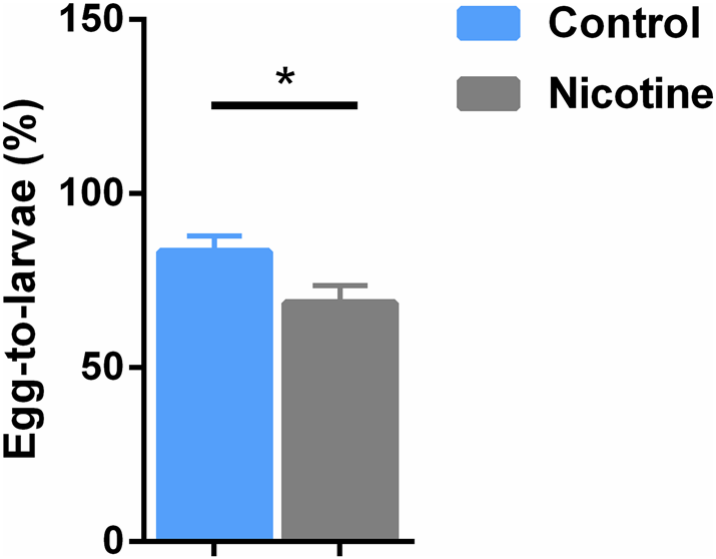
The rate of egg-to-larvae development. The percentage of egg-to-1st instar larvae development. Values are mean ± SEM, unpaired t-test, **p ≤ 0.05, n = 3 independent experiments.

As the reduced number and size of the laid eggs is induced by the direct nicotine toxicity, we followed the survival of the deposited eggs, i.e. the egg-to-larvae development. There was a 20% (p < 0.05) decrease in the percentage of egg hatchability and therefore their development to larva following the maternal e-nicotine exposure in comparison to the percentage of larvae which hatched from the eggs laid by the control group (Fig. 3).

### The effect of prenatal e-nicotine exposure on the growth of larva at different developmental stages

**Figure 4:**
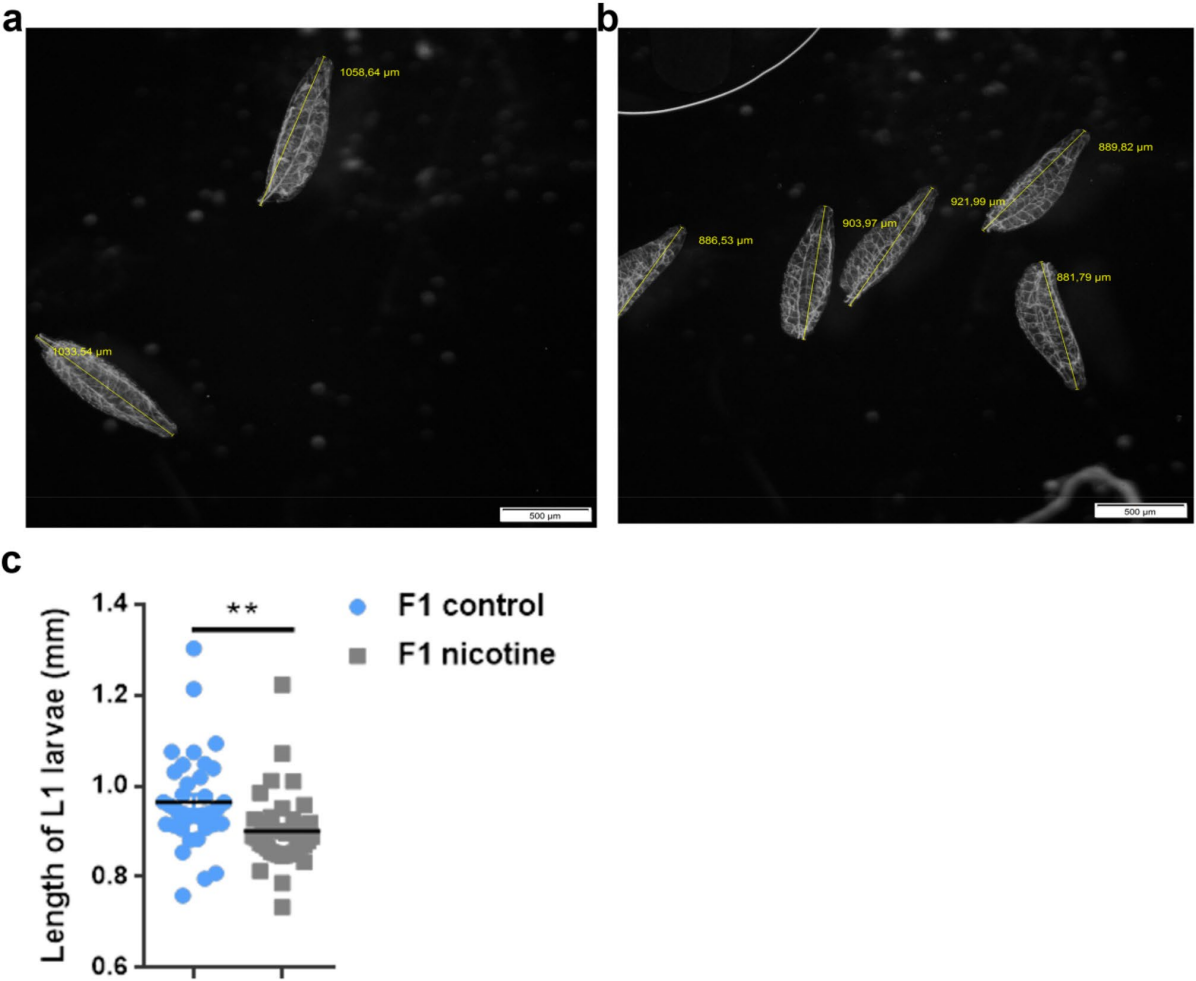
The length of F1 1st instar larvae in the control and nicotine-exposed groups. (**a**,**b**) Stereomicroscope image of the 1st instar larvae from the control (**a**) and maternal nicotine-treated (**b**) groups analysed using the image software cellSens (yellow line represents the measurement of the body length) (**c**) Quantification of the length (μm) of 1st instar larvae from maternal nicotine-treated (gray) and control (blue) groups. Values are mean + /-SEM, unpaired t-test, **p < 0.01, n = 3 independent experiments. Bar represents 500 μm.

In order to analyze whether maternal e-nicotine exposure affects the size of the hatched F1-generation larvae, the length of 1st instar larvae was measured 24 h post-hatching and compared to water-treated controls. The average body length of F1 1st instar larvae from mothers who had been exposed to e-nicotine (Fig. 4b,c; right column) was decreased by around 100 μm in comparison to the control groups (Fig. 4a,c; left column).

**Figure 5:**
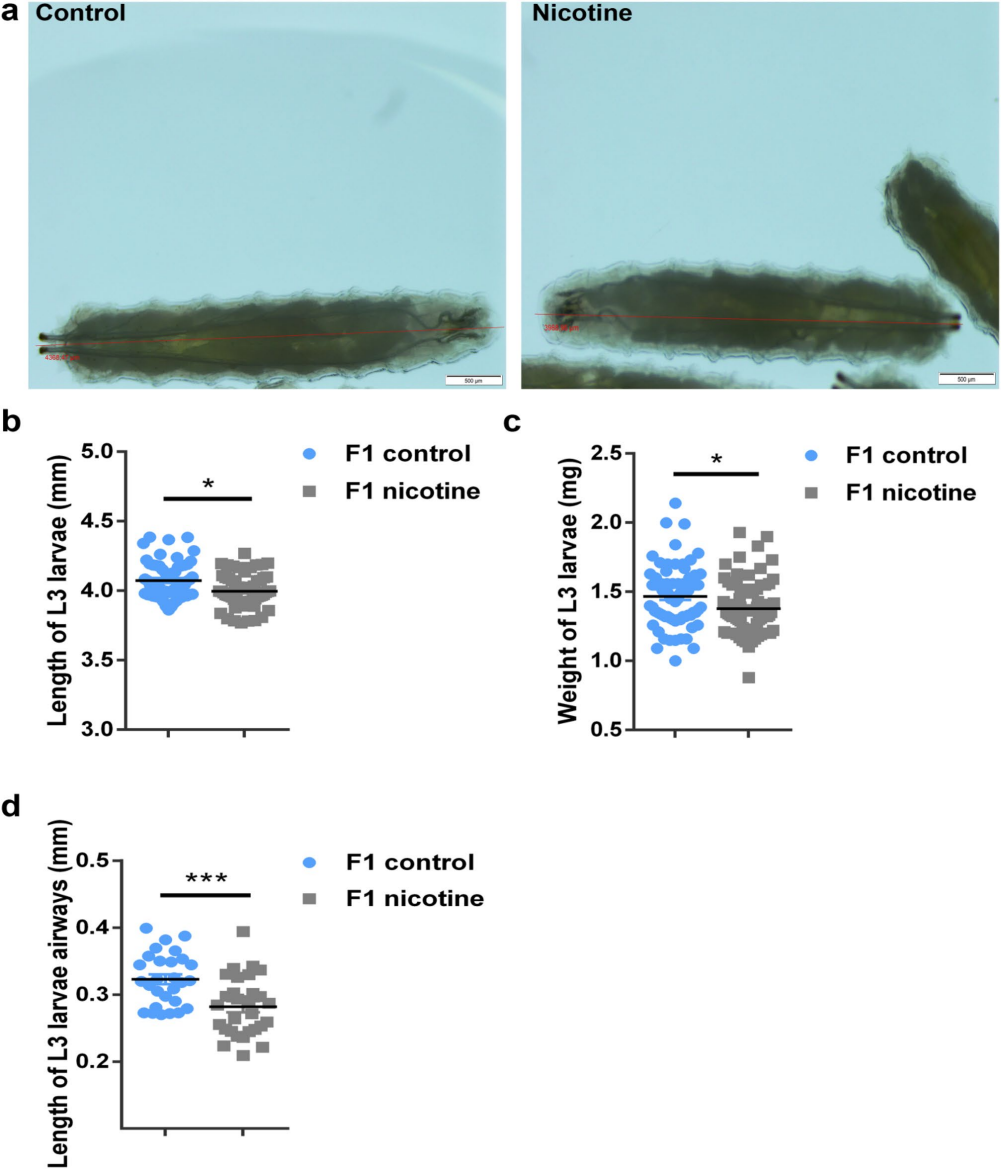
The length, weight and the extent of the airways of F1 3rd-instar larvae. (**a**) Stereomicroscope image of 3rd-instar larvae from the maternal nicotine-treated (gray) and control (blue) groups (red line represents the measurement of the body length) (**b**) Quantification of the 3rd-instar larvae body length (mm) (**c**) Weight of F1 3rd-instar larva (mg) from the control and e-nicotine-exposed mothers (**d**) Length (mm) of the L3 larvae airways measured using a stereomicroscope analysed using the image software cellSens. Values are mean ± SEM, unpaired t-test, *p < 0.05, ***p < 0.001, n = 3 independent experiments. Bar represents 500 μm.

This growth reduction was maintained until the 3rd-instar larval stage (p < 0.01) (Fig. 5a,b). Moreover, the weight of the 3rd-instar larvae from the e-nicotine exposed mothers (Fig. 5c; right column) was significantly decreased (p < 0.01) as compared to the water controls. No other obvious morphological anomalies were observed. Since maternal tobacco smoking is related to lung function deficits, we measured the tracheal length of L3 larvae which was significantly reduced in comparison to the control L3 larvae (Fig. 5d) which nonetheless did not affect lifespan (see Supplementary Fig. S1). To obtain first insight into the mechanisms underlying the reduced larval airway length, we analyzed the expression level of genes involved in forming the tracheal tubes from early embryonic stages throughout larval life^37–41^. Although these genes are major players in length development of the *Drosophila* trachea, none of the investigated genes showed altered expression by maternal e-nicotine exposure (see Supplementary Fig. S2 (female larvae), Fig. S3 (male larvae)).

### Effect of maternal e-nicotine-exposure on the size and weight of adult female and male flies

**Figure 6:**
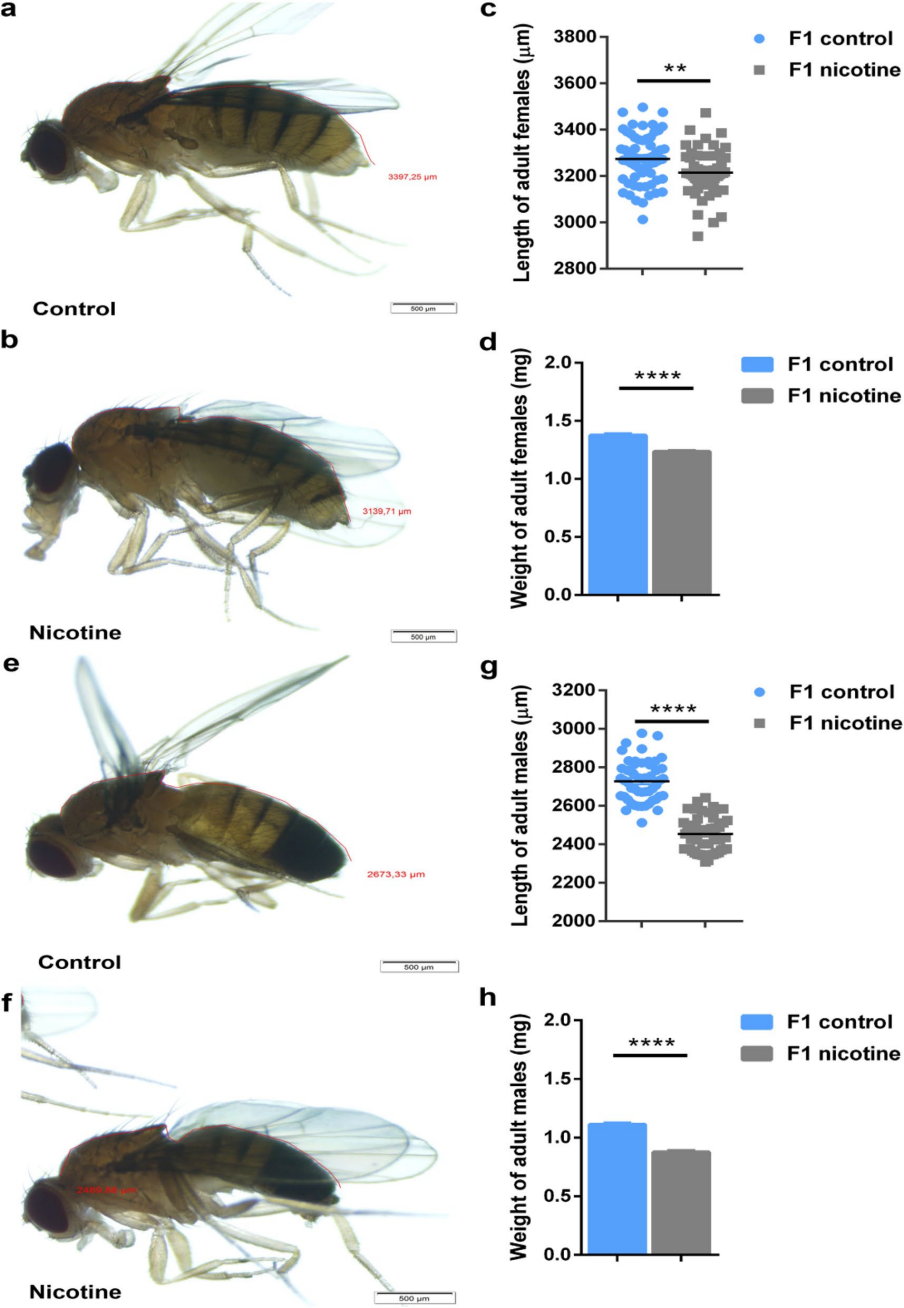
The length and weight of F1 adult flies. (**a**,**b**) Stereomicroscope image of the F1 female adult flies from the maternal e-nicotine-treated (**b**) and control (**a**) groups. (**c**,**d**) quantification of the length (μm) (**c**) and weight (mg) (**d**) of F1 adult female flies. (**e**,**f**) Stereomicroscope images of F1 male adult flies from the maternal e-nicotine-treated (**f**) and control (**e**) groups analysed using the image software cellSens. (**g**,**h**) quantification of the length (μm) and weight (mg) of the adult male flies. (**a**,**b**,**e**,**f**) red line represents the measurement of the adult fly body length. Values are mean ± SEM, unpaired t-test, **p = 0.001, ****p < 0.0001, n = 3 independent experiments. Bar represents 500 μm.

Since the reduced length and weight of L3 larvae were unlikely to still reflect direct nicotine toxicity, we hypothesized that the growth reduction could be sustained in adult flies. Indeed, maternal exposure to e-nicotine induced a reduction in the average body length of 5-day old F1 virgin female flies by around 80 μm (p < 0.01) (Fig. 6b,c; right column) as compared to the flies from the control group (Fig. 6a,c; left column). Similarly, we observed a decrease in the weight of these F1 female flies from around 1.4 mg in the control group (Fig. 6d; left column) to 1.2 mg in the maternal e-nicotine-treated groups (p < 0.0001) (Fig. 6d; right column).

Similar to female F1 virgins, the average body length of F1 adult male flies was decreased by approximately 270 μm (p < 0.0001) (Fig. 6f,g; right column) as compared to the flies from the control groups (Fig. 6e,g; left column). Of note this decrease was more pronounced as compared to F1 females. In line, the body weight of these F1 male flies decreased from approximately 1.1 to 0.8 mg in the maternal-treated groups (p < 0.0001) (Fig. 6h, right column).

### Weight to length ratio in F1 adult flies

**Figure 7:**
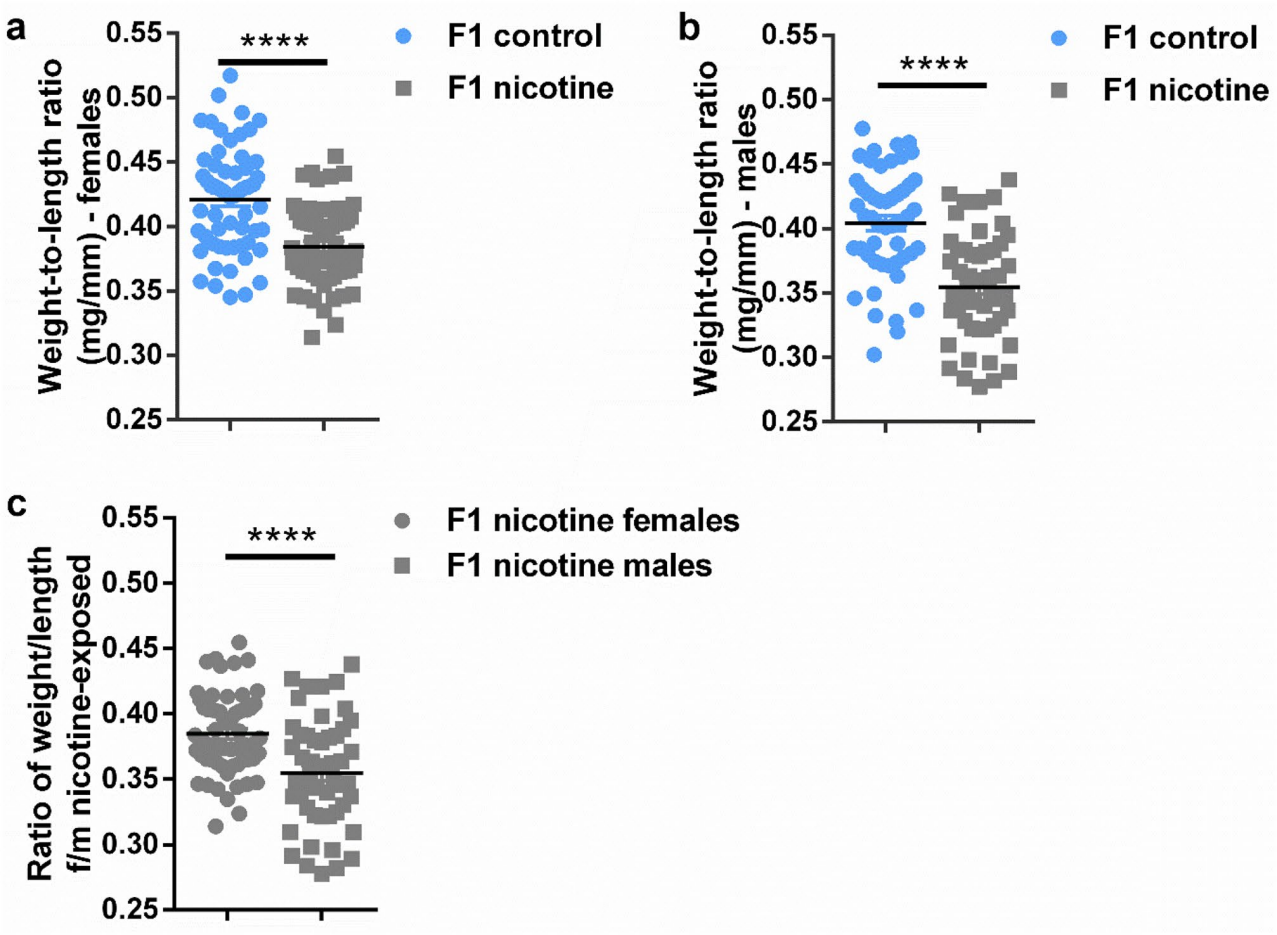
Ratio of weight-to-length in F1 adult flies. (**a**,**b**) Ratios of weight-to-length in F1 females (**a**) and males (**b**) following the maternal nicotine exposure as compared to the control group. (**c**) ratio of weight-to-length in females/males following the exposure to the maternal e-nicotine only. Values are mean ± SEM, unpaired t-test, ****p < 0.0001, n = 3 independent experiments.

Since the size of F1 male seemed to be strongly affected by maternal e-nicotine as compared to F1 females, we hypothesized that maternal e-nicotine exposure could affect both sexes differently and therefore calculated weight-to-length ratios (Fig. 7a,b). Indeed the difference in the weight/length ratios of the F1 adult females from the control and e-nicotine group was smaller (mean ± SEM 0.0363 ± 0.00659) as compared to the male flies (mean ± SEM 0.0495 ± 0.00839) (Fig. 7c) indicating that prenatal e-nicotine exposure affects males more negatively than females.

## Discussion

The adverse health effects of prenatal exposure to tobacco smoke has been recognized for many years^42–50^. Therefore, women are turning to other forms of nicotine, the electronic nicotine delivery systems, with the perception of relative safety of e-cigarettes^51–53^. In spite of this, the knowledge about the hazardous prenatal effects of e-cigarettes and their effect on early human development has received undue limited attention. Moreover, studies on the effects of e-cigarette exposure on the intergenerational health could span generations. Thus, a model to study the postnatal outcomes of prenatal e-cigarette exposure is warranted and critically important for safety surveillance of any nicotine-containing product.

Our data demonstrate that females exposed to e-nicotine exhibit nicotine sensitivity where their ability to climb in a negative geotaxis assay was impaired in a dose dependent manner. Moreover, they exhibit a reduced fertility as shown by a lower egg-deposition. In addition, eggs and hatching larvae from the e-nicotine-exposed mothers have shorter length. These findings may reflect direct effect of e-nicotine on the female organism. However, flies undergo a marked metamorphosis during pupation, in which organs and tissues are completely rebuilt from stem cells^54^. Thus, any changes beyond pupation induced by maternal e-cigarettes cannot be explained by acute toxicity.

Climbing is an innate behavior in which fruit flies climb the wall due to their natural tendency for negative geotaxis^36^. The behavior that we observed in female flies following nicotine-volatilization is in accordance with the previous studies which showed that acute exposure of *Drosophila* to nicotine impairs their climbing behavior as well as induces their hyperactivity and spasmodic movements^36,55,56^.

Borkovec (1966), reported that the decrease in fecundity following the exposure could be induced by rapid absorption of the compound through the cuticle thus affecting gonadal cells of the young flies which are in a state of division^57^. Several meta-analyses have shown that smoking reduces fertility among women^58–60^. Despite the increasing prevalence of e-cigarette use, the data regarding the effects of e-cigarettes on female fertility are lacking^1^. Even if fetuses were exposed to fewer toxins through e-cigarettes as compared to tobacco, the exposure to nicotine still causes measurable adverse fertility and pregnancy outcomes^61,62^. Moreover, there is also a limited number of experimental studies showing the effects of prenatal e-cigarette exposure in rodents. A recent study in female mice demonstrated that e-cigarette use delayed embryo implantation, thus delaying and reducing fertility^18^. Moreover, maternal exposure to e-nicotine was shown to adversely influence the structure and the physiology of the oviduct thus impairing the likelihood of pregnancy^63–65^. Therefore, prenatal exposure to nicotine compromises fetal gonadal development, affects pathways playing a role in uterine receptivity, impairs early embryo development and fertility^18,66^. Therefore it is likely that maternal e-cigs affect fertility in humans and this urgently needs to be addressed in the future studies.

We also observed a reduction in the length of the deposited eggs as well as a decrease in egg hatchability following the maternal exposure to e-nicotine. This could be attributed to the inhibitory properties of nicotine on chitin, the main component of cuticle and larval mouth-hook synthesis^67^. Nicotine could interfere with the cuticle and mouth hook development, which starts during embryogenesis, rendering them less rigid and thus depriving the larva from rupturing the egg and escaping^67^.

The size of F1 1st-instar and 3rd-instar larvae as well as the weight of the latter was significantly decreased following the exposure to maternal e-nicotine in comparison to control groups. There is considerable evidence from animal studies that perinatal exposure to e-cigarettes induces pup weight reduction^18,68,69^. Another study demonstrated that pups prenatally exposed to nicotine show delayed rate of physical maturation and growth restriction, which is in accordance with our findings showing a reduction in the length of L1 and L3 larvae following the maternal e-nicotine exposure^19,70^. The authors claim that postnatal delay in the development observed in prenatally nicotine treated pups is induced by the long lasting effect of prenatal nicotine exposure which delays motor development and physical maturation^70^. A single study in humans also showed that maternal e-nicotine might reduce birthweight, but it remained unclear if the combined use of tobacco and e-cigarettes confounded this outcome^10^.

Maternal smoking is known to affect lung function development in children^46,47,71^. Here we show that maternal e-cigarette use reduced the airway length in F1 flies indicating that maternal e-cigarette use impairs early airway development.

To identify which developmental genes are regulated by maternal nicotine and could thus explain the reduced airway length of Drosophila larvae we investigated known candidates that are involved in forming the tracheal tubes from early embryonic stages throughout the larval life^37–41^. Forming the correct tracheal length requires an exactly equilibrated balance between these genes^37,72^. Since these genes did not showed any difference in response to maternal e-nicotine, we assume that the dysregulation either occurs earlier during development or is more complex (see Supplementary Fig. S2 and S3).

A limitation of our study is the difficulty to estimate how the dose of e-nicotine applied relates to human exposure. Although the fly has homologues of the human cytochrome P450 CYP1A1 which is necessary for xenobiotic metabolism, the cotinine levels in the fly remained below detection limits (data not shown). Despite reduced airway length, F1 generation showed a normal lifespan (see Supplementary Fig. S1), therefore indicating that maternal exposure was not overly strong. Moreover, maternal life span was not reduced as opposed to stronger smoke or nicotine exposure models^73,74^ indicating that these are subthreshold concentrations for mortality with significant physiological effects.

We show here for the first time that maternal exposure to nicotine resulted in decreased weight gain and a reduction in body size even in adult females and males of the F1 generations in *D. melanogaster*. Therefore, our data suggest that maternal e-cigarette use can modulate long-term health outcomes in offspring. Our results are consistent with murine studies showing that prenatal exposure to nicotine affects metabolic function in F1 adult mice^18^ and that prenatal exposure to e-cigarettes influences body weight gain through genetic and epigenetic programming^20,75,76^.

The body weight-to-length ratio differed between female and male flies of F1 generation showing increased vulnerability of male offspring to maternal e-nicotine. This could be attributed to the fact that female *Drosophila* flies exhibit higher oxidative stress resistance as well as possess higher antioxidant defense mechanism in comparison to male flies and therefore could tolerate maternal nicotine exposure much better^77^. In line with this, maternal smoking triggers changes in DNA methylation resulting in methylation differences that are more apparent in male offspring^78,79^. Therefore, it is possible that epigenetic modifications induced by environmental stressors may at least in part be more apparent in males who show higher susceptibility to toxins than females^79–81^.

Our results demonstrate that maternal exposure to e-cigarette is associated with fetal abnormalities that persist into adulthood in flies. Currently, there are no prospective epidemiological studies in humans that examine long-term effects of maternal e-cigarette smoke exposure on the health of the offspring until adulthood and across generations. We hope that our results underline the need for human studies investigating the effects of maternal vaping on the respiratory health of their offspring.

## Supplementary Information

Supplementary Information
