# Supplementary material for: Sex dependent effect of maternal e-nicotine on F1 Drosophila development and airways
Source: Sci Rep. 2021 Feb 24;11:4441. doi: 10.1038/s41598-021-81607-8 (PMC7904947; doi:10.1038/s41598-021-81607-8)
Supplement: Supplementary file 1 — Supplementary Information [file 41598_2021_81607_MOESM1_ESM.docx]

**Supplementary Information**

Sex dependent effect of maternal e-nicotine on F1 *Drosophila* development and airways

Natalia El-Merhie^1,6*^, Arne Kruger^1*^, Karin Uliczka^1,2^, Stephanie Papenmeier^1,2^, Thomas Roeder^3,6^, Klaus F. Rabe^4,6^, Christina Wagner^2^, Hanna Angstmann^#1,6^, Susanne Krauss-Etschmann^#1,5,6^

^1^Division of Experimental Asthma Research, Early Life Origins of Chronic Lung Disease, Research Center Borstel, Leibniz Lung Center, Borstel, Germany

^2^Invertebrate Models, Priority Area Asthma & Allergy, Research Center Borstel, Leibniz Lung Center, Borstel, Germany

^3^Departments of Molecular Physiology and Zoology, Christian Albrechts University Kiel, Kiel, Germany

^4^Department of Pneumology, LungenClinic, Grosshansdorf, Germany; and Department of Medicine, Christian Albrechts University, Kiel, Germany

^5^Institute for Experimental Medicine, Christian-Albrechts-Universität zu Kiel, Kiel, Germany

^6^Member of the German Center for Lung Research (DZL) and the Airway Research Center North (ARCN)

*^#^Shared authorships

**Corresponding author**

Susanne Krauss-Etschmann, MD

Early Life Origins of Chronic Lung Diseases

Research Center Borstel

Leibniz Lung Center

Parkallee 1-40

D-23845 Borstel

Tel. +49 (0)4537 188-5850

[skrauss-etschmann@fz-borstel.de](mailto:skrauss-etschmann@fz-borstel.de)

Airway Research Center North (ARCN)

Member of the German Center for Lung Research (DZL)

**Supplementary methods**

**Molecularbiological analyses**

The isolation and purification of RNA from dissected larval tracheae was performed with the NucleoSpin RNA II Kit (Macherey-Nagel) according to the manufacture’s protocol. For reverse transcription of RNA into cDNA, 250ng of total RNA was mixed with 200u SuperScript III (Thermo Fischer Sci.) in a final volume of 20µl.

The reaction mixture was incubated in the thermal cycler for one hour at 50°C and the reaction was inactivated at 70°C for 15 min. Before use in real-time PCR, the cDNA samples were diluted with an equal volume of RNAse-free H_2_O.The quantification of gene expression was measured in a real-time PCR (qPCR) using the Lightcycler 480 (Roche). The reaction mixture consisted of SYBR Green Mastermix (Roche), 10 µM oligonucleotides and 2 µl cDNA. *coracle*: f: 5’-AAATCAGCCAACCGCCAAAC-3’, r: 5’-CGGCAATGATTTCCAGCGAG-3’; *src oncogene at 42A*: f: 5’-GAAGCCTGTAACTGAGGGGC-3’, r: 5’-CGGCAATGATTTCCAGCGAG-3’; *pointed transcript variant RB, RD*: f: 5’-TTGGACATCCTGCAAAAAGA-3’, r: 5’-GGTGGTCCGATCCACAGA-3’; *pointed transcript variant RC, RE*: f: 5’-TCGGATGTCAACTTCTTTAGTTCA-3’, r: 5’-GCTACCGCTGCCATTGAC-3’; pickle: f: 5’-TCGAAAAGGGCGACTATCC-3’, r: 5’-AGCTGGCGAATGTAACTATGG-3’; *crumbs*: f: 5’-CCGCTCTTCATACTTATTTACTTAGCA-3’; r: 5’-GGAGGTAAGTGGAGCCATTAAA-3’; *breathless transcript variant RB*: f: 5’-GCGCACATTCAAGCCAAT-3’, r: 5’-TCAAATCGAGCGTTCTCCA-3’; *ribosomal protein L32*: f: 5’-CCAGTCGGATCGATATGCTAA-3’, r: GTTCGATCCGTAACCGATGT-3’.

**Supplementary results**

**
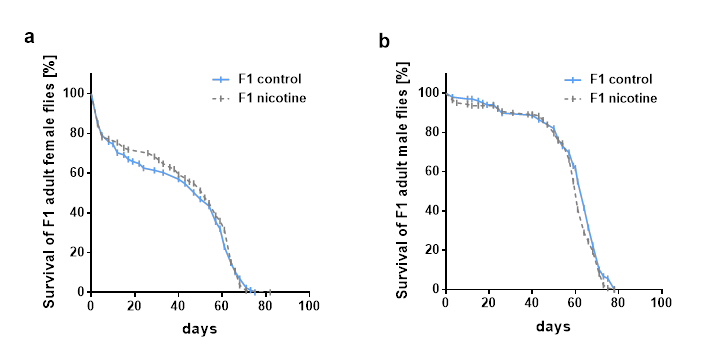
**

**Supplementary Fig. S1. Survival of F1 *Drosophila* flies following nicotine exposure.**

Percentage of living control (blue) or nicotine treated (grey) F1 females (a) and F1 males (b). Kaplan-Meier curve, log-rank (Mantel-Cox) test, n = 4 experiments, 100 animals in total.


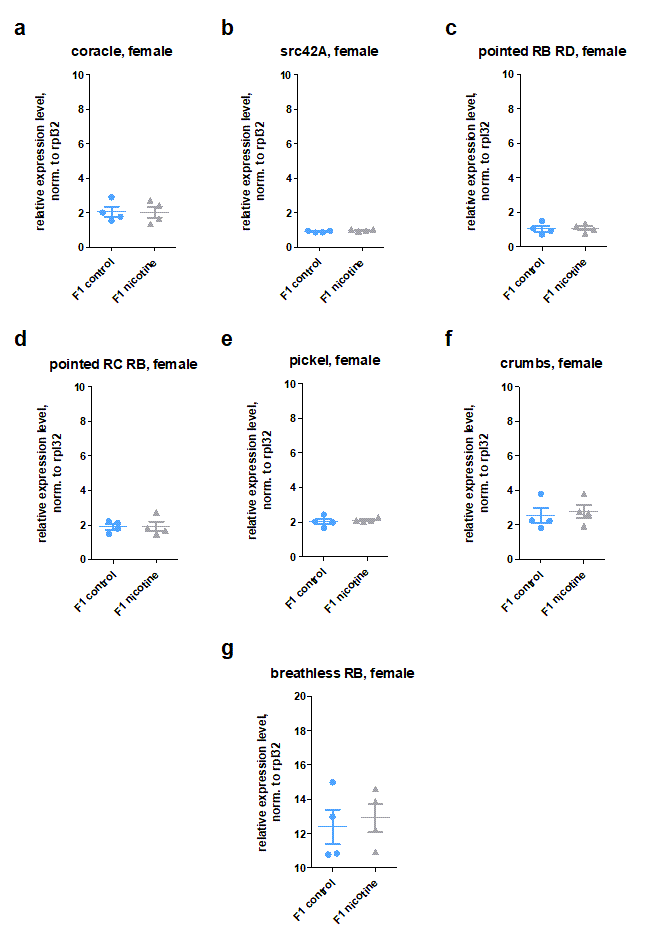


**Supplementary Fig. S2. Expression levels of relevant genes for airway development in isolated female larval tracheae.**

Relative expression levels normalized to *rpL32* in isolated female larval tracheae for *coracle* (a), *src42* (b), *pointed transcript variant RB, RD* (c), *pointed transcript variant RC, RB* (d), *pickel* (e), *crumbs* (f), *breathless transcript variant RB* (g). Mean ± SEM, ratio-paired t-test., n = 4 experiments, 40 dissected tracheae per sample.


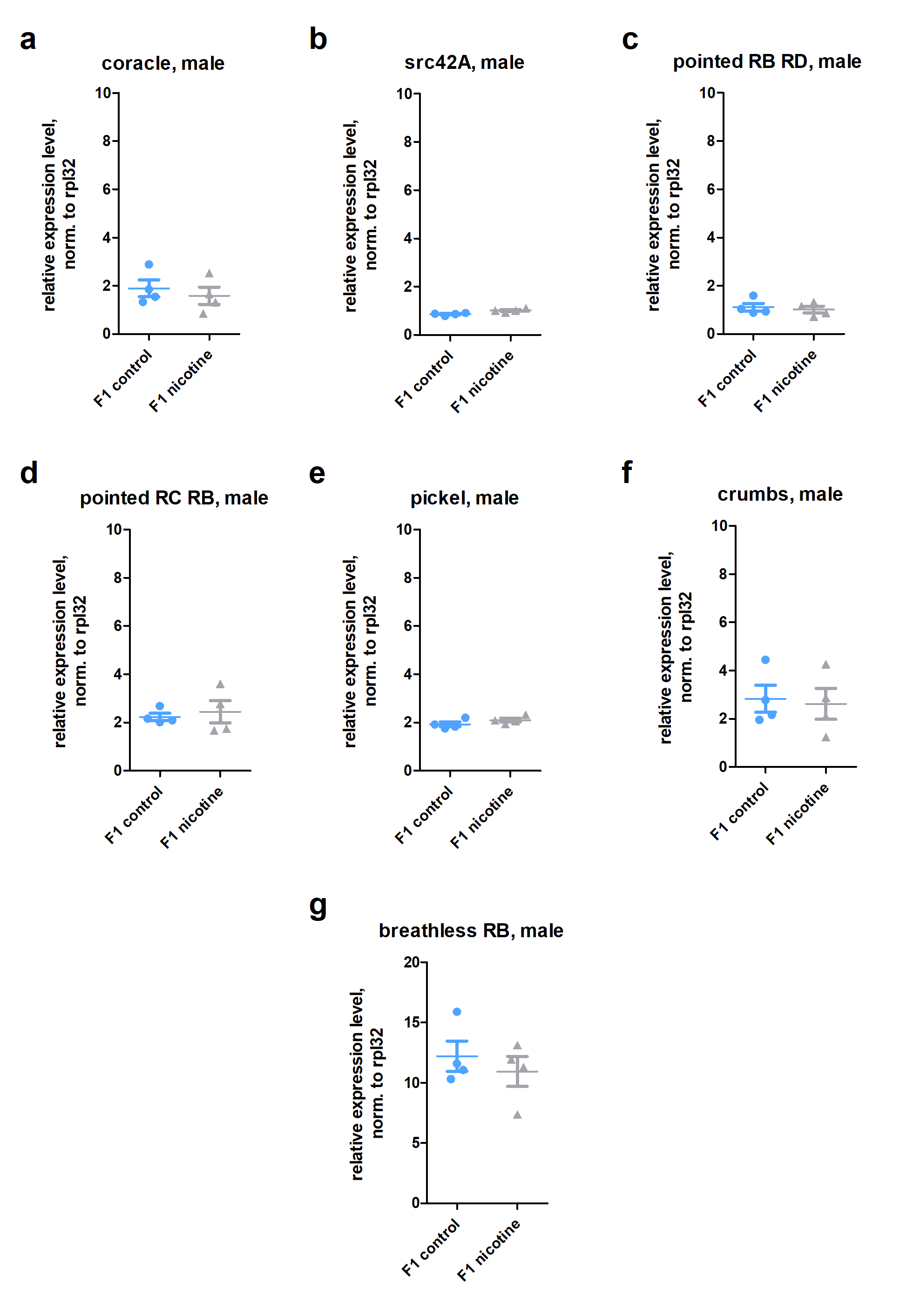


**Supplementary Fig. S3. Expression levels of relevant genes for airway development in isolated male larval tracheae.**

Relative expression levels normalized to *rpL32* in isolated male larval tracheae for *coracle* (a), *src42* (b), *pointed transcript variant RB, RD* (c), *pointed transcript variant RC, RB* (d), *pickel* (e), *crumbs* (f), *breathless transcript variant RB* (g). Mean ± SEM, ratio-paired t-test., n = 4 experiments, 40 dissected tracheae per sample.
